# Evaluating the Feasibility and Safety of Daily Inter-facility Travel for Proton Beam Therapy During Chemotherapy-Induced Myelosuppression in Children With Cancer

**DOI:** 10.7759/cureus.103052

**Published:** 2026-02-05

**Authors:** Yu Furui, Masayuki Araya, Hidekazu Masaki, Naoto Mizushiro, Keiichiro Koiwai, Eriko Uchida, Hirokazu Morokawa, Eri Okura, Kazutoshi Komori, Daisuke Morita, Takashi Kurata, Koichi Hirabayashi, Shoji Saito, Miyuki Tanaka, Yozo Nakazawa, Kazuo Sakashita

**Affiliations:** 1 Department of Hematology and Oncology, Nagano Children's Hospital, Azumino, JPN; 2 Department of Pediatrics, Shinshu University School of Medicine, Matsumoto, JPN; 3 Department of Radiology, Center of Proton Therapy, Aizawa Hospital, Matsumoto, JPN; 4 Department of Pediatrics, Aizawa Hospital, Matsumoto, JPN; 5 Department of Radiology, Shinshu University School of Medicine, Matsumoto, JPN; 6 Department of Hematology and Oncology, Nagano Children’s Hospital, Azumino, JPN

**Keywords:** outpatient treatment, patient transfer, pediatric cancer, proton therapy, radiation therapy

## Abstract

Introduction: Proton beam therapy (PBT) is widely used in pediatric oncology to reduce radiation exposure to normal tissues and mitigate late adverse effects. In Japan, the limited availability of centers that can provide pediatric PBT with sedation and/or concurrent chemotherapy often necessitates inter-facility travel. However, the safety of daily inter-facility travel for PBT during chemotherapy-induced myelosuppression has not been well evaluated. We assessed the feasibility and safety of this model in Nagano Prefecture.

Methods: We conducted a retrospective cohort study using data from three hospitals in Nagano Prefecture (Nagano Children’s Hospital, Shinshu University Hospital, and Aizawa Hospital) from June 2011 to August 2021. Patients who underwent PBT at a proton center with daily inter-facility travel during chemotherapy-induced myelosuppression were compared with patients who received conventional X-ray therapy (XRT) without transfer. Myelosuppression was defined as an absolute neutrophil count <500/µL. We extracted data on infection prophylaxis, febrile neutropenia (FN), documented infections, radiotherapy interruptions, and other adverse events, including events related to inter-facility travel or sedation.

Results: Among 166 pediatric patients who received radiotherapy, 55 underwent PBT and 111 underwent XRT. Of these, 20 patients in the PBT group and 18 in the XRT group developed myelosuppression during concurrent chemoradiotherapy and were included in the analysis. Treatment interruptions were more frequent in the PBT group, but most lasted one to two days. FN was the most common immediate reason for interruption. There were no significant differences between groups in the incidence of FN or documented infections, and no adverse events were clearly attributable to inter-facility travel or sedation in the PBT group.

Conclusions: In this cohort, daily inter-facility travel for pediatric PBT during chemotherapy-induced myelosuppression was feasible. Although brief treatment interruptions were more frequent in the PBT group, infectious outcomes were comparable to those of patients receiving XRT without transfer, and no travel- or sedation-related adverse events were identified. Coordinated multidisciplinary protocols may help minimize avoidable interruptions while maintaining safety.

## Introduction

Proton beam therapy (PBT) is a conformal form of radiotherapy that can substantially reduce the integral dose delivered to surrounding normal tissues compared with conventional photon-based radiotherapy [1-3]. In children, where tissues are developing and long-term survival is common, reducing dose to organs at risk is clinically important because late toxicities may include endocrine dysfunction, growth impairment, neurocognitive sequelae, cardiopulmonary toxicity, and second malignancies [3,4]. For these reasons, PBT has become an important component of multidisciplinary pediatric cancer care [1-5].

In Japan, pediatric PBT has been reimbursed since 2016 [4]. Despite increasing availability, only a subset of centers can provide pediatric PBT under sedation and/or while coordinating concurrent chemotherapy. As a result, some patients must receive chemotherapy at their primary hospital and travel to a separate proton center for daily irradiation. Several groups have reported that inter-institutional transfer to access PBT is feasible in children, including cases of intracranial rhabdomyosarcoma, Ewing sarcoma family tumors, and high-risk neuroblastoma [5-8]. However, in most prior reports, transfer was arranged after recovery from post-chemotherapy marrow suppression or between chemotherapy cycles, rather than during active myelosuppression [6-8].

Daily transfer during chemotherapy-induced myelosuppression raises specific safety concerns, particularly infection risk. Febrile neutropenia (FN) is among the most frequent and clinically important complications of chemotherapy in children; contemporary reviews estimate that roughly one-third of pediatric patients receiving chemotherapy experience FN during treatment courses, although the incidence varies widely by diagnosis and regimen [9]. Current international pediatric FN guidelines emphasize rapid evaluation, early empiric antibiotics, and risk-adapted supportive care [10]. In addition to infection risk, treatment interruptions are a practical concern: prolonged radiotherapy duration has been associated with inferior outcomes in some pediatric CNS tumors, particularly in higher-risk settings [11]. Therefore, understanding whether daily transfer during myelosuppression increases infectious complications and/or interruptions is clinically relevant.

In Nagano Prefecture, three institutions have established a shared-care system in which pediatric patients receive inpatient chemotherapy at their primary hospital and undergo PBT at a proton center via daily, medically supervised inter-facility transport when needed. The present study aimed to evaluate the feasibility and safety of this model during periods of chemotherapy-induced myelosuppression. The primary endpoint was any radiotherapy interruption during the course, and secondary endpoints included interruption duration, FN, documented infections, and adverse events related to inter-facility travel or sedation.

## Materials and methods

This retrospective cohort study used clinical data from Nagano Children’s Hospital, Shinshu University Hospital, and Aizawa Hospital. The study period was June 2011 to August 2021. During this period, pediatric patients receiving chemotherapy were generally hospitalized during myelosuppression for close monitoring and supportive care. We identified all pediatric patients who received radiotherapy at the participating institutions during the study period. The PBT group included patients treated with PBT at the Aizawa Hospital Proton Therapy Center (January 2016-August 2021), with chemotherapy administered at Nagano Children’s Hospital and/or Shinshu University Hospital. The X-ray therapy (XRT) group included patients treated with conventional X-ray therapy at Nagano Children’s Hospital (June 2011-August 2021). The longer inclusion period for the XRT group was used to secure an adequate number of patients who received concurrent chemotherapy and developed myelosuppression for comparison.

Inclusion criteria for the analytic cohort were (1) radiotherapy delivered with concurrent chemotherapy and (2) chemotherapy-induced myelosuppression during the radiotherapy course. Myelosuppression was defined a priori as an absolute neutrophil count <500/µL. Exclusion criteria were (1) intensive care unit management at the initiation of radiotherapy (one patient excluded) or (2) insufficient medical record data for the primary outcomes. Patients in the XRT group did not undergo inter-facility transfer and served as the control group. Patients in the PBT group underwent inter-facility transfer for irradiation and served as the comparison group.

Radiotherapy was delivered on weekdays. PBT was provided as an outpatient visit to the proton center while patients remained under the care of their primary hospital. Patients with neutrophil counts ≥500/µL could travel by private car. Patients with neutrophil counts <500/µL, those receiving chemotherapy, or those with poor general condition were transported using a designated medical transport vehicle accompanied by a nurse. To reduce infection risk during transport, patients and staff wore masks, the vehicle was disinfected before and after transport, and contact with other patients was avoided. The medical vehicle was not equipped with a high-efficiency particulate air (HEPA) filter. Travel distances were approximately 4 km (10 minutes) between Shinshu University Hospital and Aizawa Hospital, and approximately 10 km (30 minutes) between Nagano Children’s Hospital and Aizawa Hospital.

Sedation for irradiation was performed mainly by pediatricians with intensive care experience in cooperation with anesthesiologists at Aizawa Hospital. Repeated anesthesia/sedation for pediatric radiotherapy, including proton therapy, has been reported as feasible when delivered under structured protocols [12,13]. Decisions to delay transfer and/or irradiation were made by the treating team based on clinical status (e.g., suspected or confirmed FN, anemia in patients requiring sedation, or poor general condition), prioritizing safety.

We extracted the following variables from medical records: age, sex, diagnosis, prophylactic antibacterial/antifungal use, severity of leukopenia, duration of neutropenia, occurrence of FN, fungal infection, viral infection, number and duration of radiotherapy interruptions, and other adverse events. Adverse events were graded according to the Common Terminology Criteria for Adverse Events (CTCAE) version 5.0.

The primary outcome was any radiotherapy interruption during the course. Secondary outcomes included interruption duration, FN incidence, documented fungal/viral infections, and adverse events associated with transfer or sedation.

This was an exploratory retrospective study; therefore, no a priori sample size calculation was performed. We included all eligible patients within the study period. As a post hoc assessment, with n=20 (PBT) and n=18 (XRT), the study had adequate power to detect large between-group differences in the primary endpoint (treatment interruption). In contrast, the study was underpowered to detect modest differences in less frequent outcomes such as FN; therefore, null findings for FN/infections should be interpreted cautiously.

Statistical analyses were performed using EZR (Easy R), a graphical user interface for R [14]. Categorical variables were compared using Fisher’s exact test. All p-values were two-sided, and p < 0.05 was considered statistically significant. To evaluate the association between treatment modality (PBT versus XRT) and treatment interruption while accounting for prespecified covariates, we performed an analysis of covariance (ANCOVA).

The study protocol was approved by the ethics committees of Nagano Children’s Hospital, Shinshu University Hospital, and Aizawa Hospital. Informed consent was waived due to the retrospective design (study number S-03-8).

## Results

**Figure 1:**
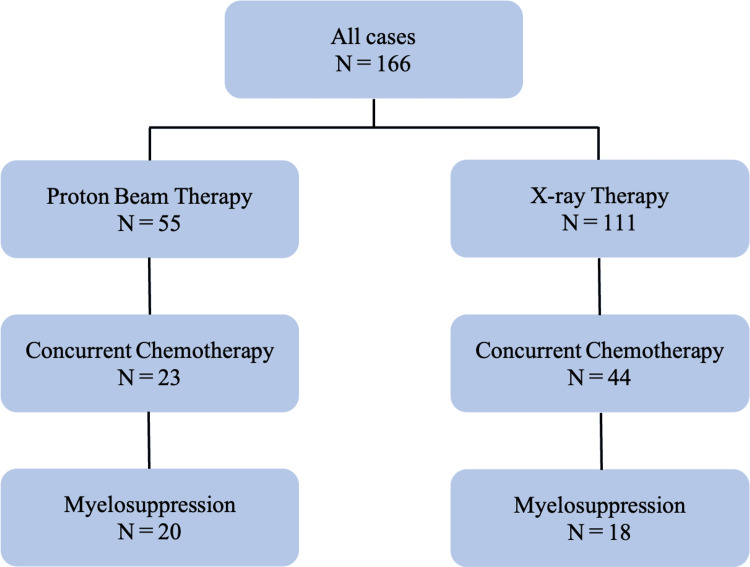
Flowchart for participant selection in research

The patient flow diagram is provided in Figure 1.

Among the patients who received radiotherapy during the study period, 20 patients in the PBT group and 18 patients in the XRT group developed chemotherapy-induced myelosuppression and were included in the analytic cohort. Baseline characteristics are summarized in Table 1.

**Table 1:** Characteristics of patients who underwent chemoradiotherapy Values are shown as number (%). P values were calculated using Fisher’s exact test. Statistical significance was set at p < 0.05. * Because of the intracranial extension of the tumor, we categorized it as a brain tumor. PBT, proton beam therapy; XRT, X-ray therapy; PNET, primitive neuroectodermal tumor; ETMR, embryonal tumor with multilayered rosettes

Characteristic	Total (n = 38)	PBT (n = 20)		XRT (n = 18)		p-value
	No.	No.	%	No.	%	
Gender						0.101
Female	21	14	70.0	7	38.9	
Male	17	6	30.0	11	61.1	
Age at chemoradiotherapy (years)						0.542
0–4	12	8	40.0	4	22.2	
5–9	9	4	20.0	5	27.8	
≥10	17	8	40.0	9	50.0	
Cancer diagnosis						<0.001
Sarcoma	20	16	80.0	4	22.2	
Rhabdomyosarcoma	18	14	-	4	-	
Ewing sarcoma	2	2	-	0	-	
Brain tumor	18	4	20.0	14	77.8	
Medulloblastoma	8	3	-	5	-	
PNET	1	0	-	1	-	
ETMR	1	0	-	1	-	
Germinoma	5	0	-	5	-	
Yolk sac tumor	1	0	-	1	-	
Ependymoma	1	0	-	1	-	
Olfactory neuroblastoma*	1	1	-	0	-	
Antibacterial prophylaxis						0.746
Yes	19	11	55.0	8	44.4	
No	19	9	45.0	10	55.6	
Antifungal prophylaxis						0.232
Yes	35	17	85.0	18	100.0	
No	3	3	15.0	0	0.0	
Severity of leukopenia (white blood cell count, /μL)						0.005
<100	13	3	15.0	10	55.6	
≥100 to <500	16	10	50.0	6	33.3	
≥500 to <1000	6	6	30.0	0	0.0	
≥1000	3	1	5.0	2	11.1	
Days of neutropenia						0.194
<7 days	13	9	45.0	4	22.2	
≥7 to <14 days	8	5	25.0	3	16.7	
≥14 days	17	6	30.0	11	61.1	
Sedation						0.004
Yes	13	10	50.0	3	16.7	
No	25	10	50.0	15	83.3	
Febrile neutropenia						0.734
Yes	12	7	35.0	5	27.8	
No	26	13	65.0	13	72.2	
Fungal infection						1.000
Yes	0	0	0.0	0	0.0	
No	38	20	100.0	18	100.0	
Viral infection						1.000
Yes	0	0	0.0	0	0.0	
No	38	20	100.0	18	100.0	
Treatment interruption						0.001
Yes	12	11	55.0	1	5.6	
No	26	9	45.0	17	94.4	
Interruption days						<0.001
0 day	26	9	45.0	17	94.4	
1–2 days	10	10	50.0	0	0.0	
≥3 days	2	1	5.0	1	5.6	

Age at chemoradiotherapy and sex distribution were similar between groups. Diagnoses differed between groups: rhabdomyosarcoma predominated in the PBT group, whereas brain tumors (including medulloblastoma and germ cell tumors) were more common in the XRT group. Supportive care and infection prophylaxis during neutropenia are summarized in Table 1. All patients received sulfamethoxazole-trimethoprim during the overall treatment period. Prophylactic antibacterial and antifungal use during neutropenia did not differ between groups. The XRT group included more patients with severe leukopenia (<100/µL), while neutropenia duration categories were comparable. Sedation for irradiation was more frequent in the PBT group. The incidence of FN did not differ between groups, and no fungal or viral infections were documented in either group. Treatment interruptions occurred more frequently in the PBT group; most interruptions lasted one to two days. An ANCOVA model evaluating factors associated with treatment interruption is shown in Table 2.

**Table 2:** Analysis of covariance (ANCOVA) results evaluating the effect of treatment type on treatment interruption, controlling for covariates Analysis of covariance (ANCOVA) was performed. Statistical significance was defined as p < 0.05. ANCOVA, analysis of covariance; FN, febrile neutropenia; PBT, proton beam therapy; XRT, X-ray therapy

Source	Sum of squares	df	F	p-value
Age at treatment (≤4 years, five to nine years, ≥10 years)	0.174	2	0.564	0.576
Classification (sarcoma, brain tumor)	0.265	1	1.717	0.201
Days of neutropenia (<7 days, seven to 13 days, ≥14 days)	0.609	2	1.977	0.159
Febrile neutropenia (FN) (+ / -)	0.504	1	3.267	0.082
Severity of leukopenia (white blood cell count: <100/μL, ≥100 to <500/μL, ≥500 to <1000/μL, ≥1000/μL)	0.405	3	0.875	0.462
Sedation ( + / -)	0.086	1	0.564	0.467
Treatment (proton beam therapy vs. X-ray radiation therapy)	1.204	1	7.813	0.010
Residual	4.008	26	-	-

Treatment modality (PBT vs. XRT) was associated with interruption, while other covariates were not statistically significant. FN showed a nonsignificant trend toward association with interruption. The details of interruptions are provided in Table 3.

**Table 3:** Summary of patients with treatment interruption CT, chemotherapy; PBT, proton beam therapy; XRT, X-ray therapy; CSI, craniospinal irradiation; Gy, gray; VAC, vincristine/actinomycin D/cyclophosphamide; VDC, vincristine/doxorubicin/cyclophosphamide; VI, vincristine/irinotecan; IE, ifosfamide/etoposide; regimen B, vincristine/cisplatin/cyclophosphamide; VPC, vincristine/pirarubicin/cyclophosphamide; VCR, vincristine; CY, cyclophosphamide; VC, vincristine/cyclophosphamide; FN, febrile neutropenia

Case no.	Sex	Age at treatment (years)	Treatment	Diagnosis	Radiation site	Radiation dose (Gy)	Fractions	Chemotherapy	Cause of treatment interruption	Interruption (days)
1	Female	7	CT + PBT	Rhabdomyosarcoma	Parameningeal	50.4	28	VAC	FN	1
2	Female	4	CT + PBT	Rhabdomyosarcoma	Left femoral	41.4	23	VAC	FN	2
3	Female	15	CT + PBT	Rhabdomyosarcoma	Parameningeal	60	30	VDC + pazopanib	FN	1
4	Male	4	CT + PBT	Rhabdomyosarcoma	Abdomen	50.4	28	VI	Drug fever	1
5	Female	18	CT + PBT	Rhabdomyosarcoma	Pelvis	50.4	34	VI, IE, VPC	Abdominal pain due to peritoneal dissemination	1
6	Female	8	CT + PBT	Medulloblastoma	CSI + Tumor bed boost	50	32	Regimen B	Anemia	3
7	Female	14	CT + PBT	Rhabdomyosarcoma	Left thorax + Lumbar spine + Right sciatic bone	50.4	28	VI, IE, VPC	FN, Radiation esophagitis	2
8	Female	6	CT + PBT	Medulloblastoma	CSI + Tumor bed boost	50	32	Regimen B	Anemia	1
9	Male	18	CT + PBT	Rhabdomyosarcoma	Left Arm	50.4	28	VI, VC	FN	1
10	Male	3	CT + PBT	Medulloblastoma	CSI + Tumor bed boost	51.2	30	Regimen B	Anemia	1
11	Female	4	CT + PBT	Rhabdomyosarcoma	Laryngeal	54	30	VCR, CY	Neutropenia	1
12	Male	2	CT + XRT	Ependymoma	Occipital tumor bed	54	30	Regimen B	FN, Bleeding	7

FN was the most common reason, followed by anemia in patients requiring sedation. For anemia-related interruptions, transfusion was performed with a target hemoglobin (Hb) ≥8 g/dL. No interruption was attributed to transfer-related events. No interruptions in the analytic cohort were attributed to radiotherapy equipment downtime. Non-hematologic grade 3-4 adverse events (excluding FN) are summarized in Table 4.

**Table 4:** Grade 3–4 non-hematologic acute adverse events observed during proton beam therapy (PBT) with concurrent chemotherapy CTCAE, Common Terminology Criteria for Adverse Events (version 5.0). PBT, proton beam therapy. Values represent the number of patients.

Adverse event	CTCAE Grade 3, n	CTCAE Grade 4, n
Radiation esophagitis	1	0
Radiation dermatitis	1	0
Oral mucositis	1	0
Febrile neutropenia	7	0

In the PBT group, grade 3 esophagitis, radiation dermatitis, and oral mucositis occurred in single cases. No toxicity was attributed to inter-facility transfer or sedation.

## Discussion

In this retrospective cohort, we evaluated a regional care model in which pediatric patients underwent PBT with daily inter-facility travel while experiencing chemotherapy-induced myelosuppression. The main findings were as follows: (1) Treatment interruptions were more frequent in the PBT group, but were usually brief (one to two days). (2) FN was the most common immediate reason for interruption. (3) There were no significant differences between groups in the incidence of FN or documented infections, and no adverse events were clearly attributable to inter-facility travel or sedation in the PBT group.

**Figure 2:**
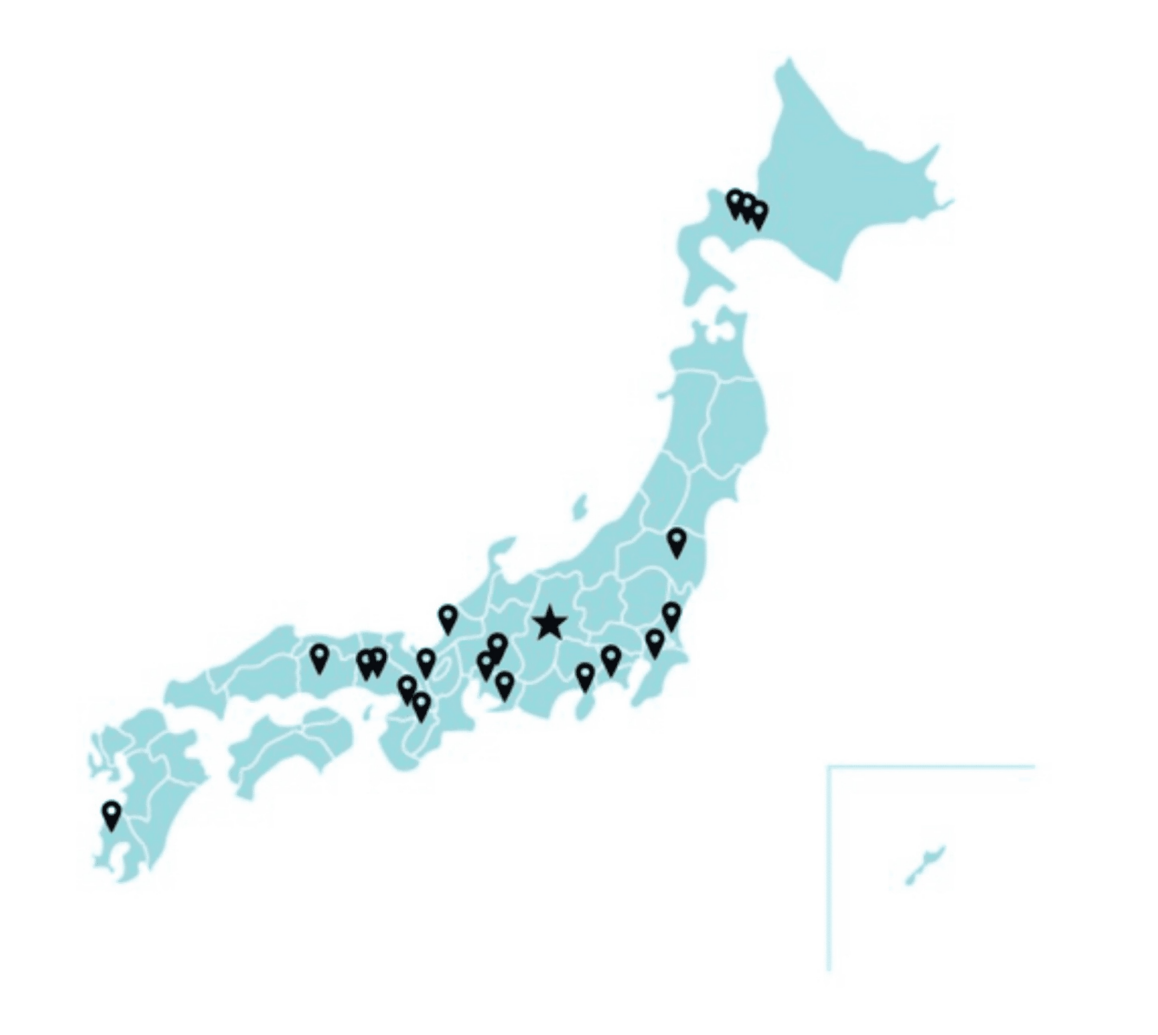
Locations of facilities capable of proton therapy in Japan Facility information was compiled from the Medical Nuclear Technology Research Promotion Foundation (accessed on January 13, 2026) [15] and plotted by the authors. The star indicates the Aizawa Hospital Proton Therapy Center.

Our results extend prior reports of inter-institutional transfer for pediatric PBT [5-8] by addressing a setting that has not been well described: daily transport during ongoing myelosuppression, with chemotherapy administered at the primary hospital. In earlier series, patients were often transferred after marrow recovery or between chemotherapy cycles [6-8]. In contrast, the current study reflects a practical solution in regions where pediatric PBT capacity is limited and where concurrent chemotherapy and sedation may be required. As of January 2026, proton therapy is available at 20 facilities nationwide in Japan (Figure 2).

However, only a subset of these centers can provide pediatric PBT while coordinating sedation and/or concurrent chemotherapy, which often necessitates inter-facility travel.

The observed pattern of interruptions likely reflects clinical risk management rather than excess toxicity. Clinicians may be more cautious about transferring patients with borderline symptoms suggestive of infection or anemia, particularly when sedation is planned. This is consistent with FN being a central driver of interruptions. FN remains common in pediatric oncology and is a major reason for hospitalization and escalation of supportive care [9,10]. Although we did not observe higher FN rates in the PBT group, the study was small and may not have detected moderate differences. Importantly, interruption duration matters. Prolonged radiotherapy duration has been associated with inferior outcomes in some pediatric CNS tumors, especially in higher-risk populations [11]. In our cohort, most interruptions lasted only 1-2 days; whether such short delays meaningfully affect tumor control depends on diagnosis, radiotherapy field, and clinical context, and should be examined in larger cohorts.

Sedation and repeated anesthesia are another practical concern in pediatric radiotherapy. Previous studies support the feasibility of repeated anesthesia/sedation for pediatric radiotherapy, including proton therapy, when delivered under standardized protocols [12,13]. In our cohort, sedation was more common in the PBT group, and anemia-related interruptions occurred only in sedated cases, reflecting a conservative approach to safety. We did not observe sedation-related grade ≥3 complications, but these events are uncommon and require larger samples to quantify.

This study has several limitations. First, the sample size was small (20 vs 18 patients), limiting statistical power, particularly for infrequent outcomes such as FN and documented infections. Second, there was substantial heterogeneity in diagnoses between groups, which could influence chemotherapy intensity, radiotherapy fields, baseline infection risk, and interruption thresholds. Third, the study periods differed between groups, introducing potential temporal confounding due to evolving supportive care practices. Fourth, the transport distances were short and medically supervised; these findings may not generalize to regions requiring longer travel times or without specialized transport infrastructure. Fifth, although basic prescription information was available, detailed radiotherapy planning parameters (e.g., target volumes and dose-volume metrics) were not systematically evaluated as predictors of interruption in this small retrospective cohort. Finally, the retrospective design limits causal inference, and we could not directly attribute or exclude transfer as a contributor to infection risk. In addition, transport logistics and supportive care practices may vary across institutions and regions, which could influence interruption thresholds and implementation of infection-control measures and thereby limit external validity.

Despite these limitations, the study provides practical evidence supporting a coordinated regional approach to pediatric PBT when on-site proton capability is unavailable and concurrent chemotherapy is required. Future multicenter studies with standardized definitions and larger samples are needed to better quantify infection risk, identify predictors of interruption, and clarify the clinical impact of short delays. Future studies should also incorporate patient-reported outcomes and family burden, including travel-related costs and time, to better characterize the overall feasibility of this care model.

## Conclusions

Daily inter-facility travel for pediatric PBT during chemotherapy-induced myelosuppression was feasible in this regional model and was not associated with excess infectious complications or transfer/sedation-related adverse events. Treatment interruptions were more frequent in the PBT group but were typically brief. Standardized protocols for inter-facility coordination, infection prevention, and sedation readiness may help minimize avoidable delays while maintaining safety.

## References

[1] MacDonald SM, DeLaney TF, Loeffler JS (2006). Proton beam radiation therapy. Cancer Invest.

[2] Rombi B, Vennarini S, Vinante L, Ravanelli D, Amichetti M (2014). Proton radiotherapy for pediatric tumors: review of first clinical results. Ital J Pediatr.

[3] Thomas H, Timmermann B (2020). Paediatric proton therapy. Br J Radiol.

[4] Mizumoto M, Fuji H, Miyachi M (2021). Proton beam therapy for children and adolescents and young adults (AYAs): JASTRO and JSPHO Guidelines. Cancer Treat Rev.

[5] Munck af Rosenschold P, Engelholm SA, Brodin PN (2016). A retrospective evaluation of the benefit of referring pediatric cancer patients to an external proton therapy center. Pediatr Blood Cancer.

[6] Inaba M, Nakao T, Hosaka S (2020). Urgent proton beam therapy with interinstitutional transfer for patients with intracranial rhabdomyosarcoma: report of 3 cases. J Pediatr Hematol Oncol.

[7] Nakao T, Fukushima H, Fukushima T (2018). Interinstitutional patient transfers between rapid chemotherapy cycles were feasible to utilize proton beam therapy for pediatric Ewing sarcoma family of tumors. Rep Pract Oncol Radiother.

[8] Hosaka S, Fukushima H, Nakao T (2020). Patient transfer to receive proton beam therapy during intensive multimodal therapy is safe and feasible for patients with newly diagnosed high-risk neuroblastoma. J Pediatr Hematol Oncol.

[9] Cennamo F, Masetti R, Largo P, Argentiero A, Pession A, Esposito S (2021). Update on febrile neutropenia in pediatric oncological patients undergoing chemotherapy. Children (Basel).

[10] Lehrnbecher T, Robinson PD, Ammann RA (2023). Guideline for the management of fever and neutropenia in pediatric patients with cancer and hematopoietic cell transplantation recipients: 2023 update. J Clin Oncol.

[11] Baliga S, Bajaj BV, Kabarriti R (2020). Prolongation of radiotherapy duration is associated with inferior overall survival in patients with pediatric medulloblastoma and central nervous system primitive neuroectodermal tumors. Pediatr Blood Cancer.

[12] Owusu-Agyemang P, Grosshans D, Arunkumar R (2014). Non-invasive anesthesia for children undergoing proton radiation therapy. Radiother Oncol.

[13] Shimazu Y, Otsuki R, Murakami M (2020). Age as a decisive factor in general anaesthesia use in paediatric proton beam therapy. Sci Rep.

[14] Kanda Y (2013). Investigation of the freely available easy-to-use software 'EZR' for medical statistics. Bone Marrow Transplant.

[15] (2026). Association for Nuclear Technology in Medicine (ANTM): introduction to particle therapy facilities in Japan [Article in Japanese]. https://www.antm.or.jp/information/clinic/.

